# Correction: Seroepidemiology of hepatitis A, B, C, D and E virus infections in the general population of Peru: A cross-sectional study

**DOI:** 10.1371/journal.pone.0250185

**Published:** 2021-04-08

**Authors:** The PLOS ONE Staff

There are errors in the images for Figs 1–5. The x-axis labels in Figs 1 and 2 and the legends in Figs 3 and 4 should be in English. The legend for Fig 5 is missing. Please see the correct Figs 1–5 here.

**Fig 1:**
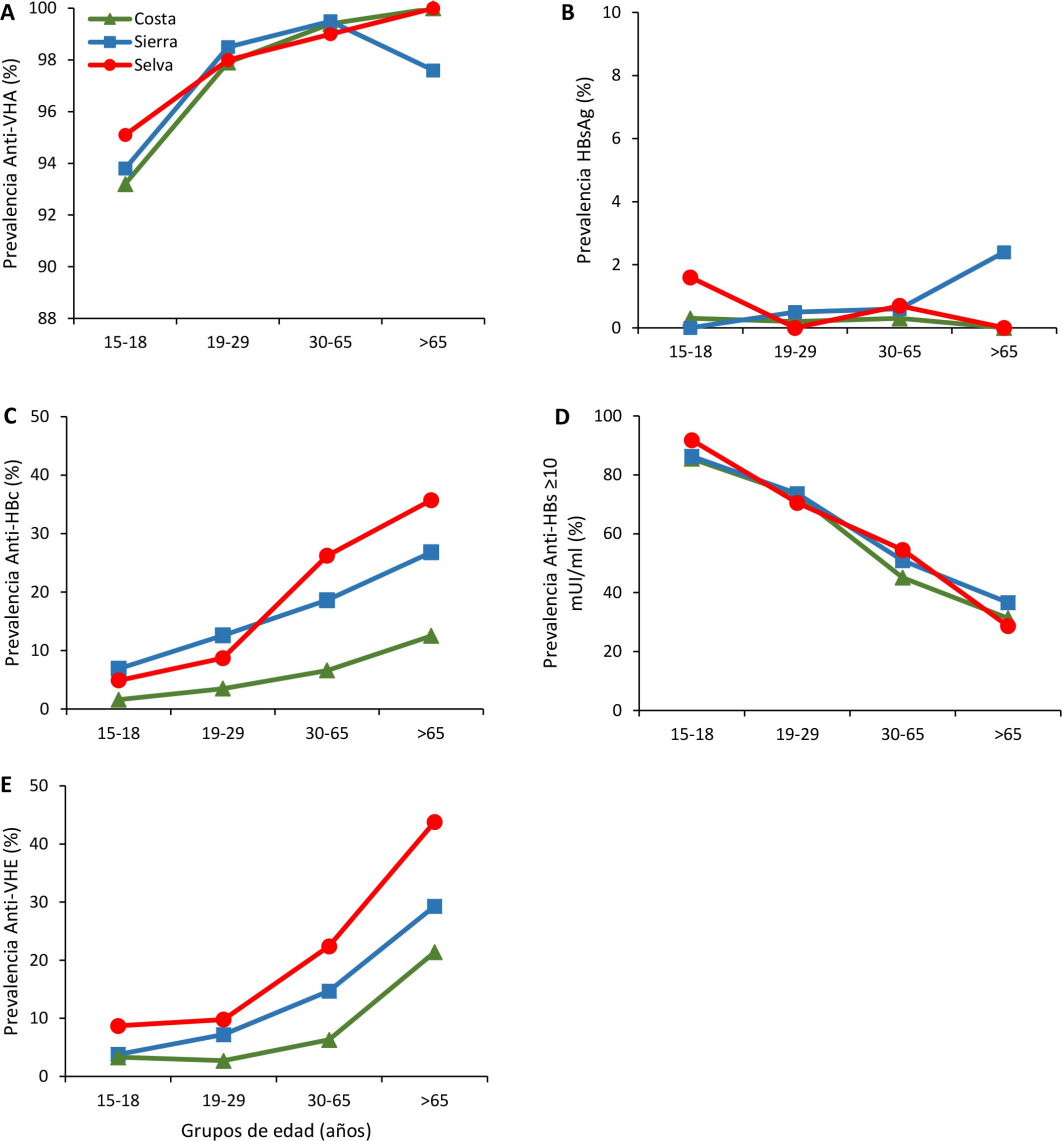
Prevalence rates of anti-HAV IgG (A), HBsAg (B), anti-HBc IgG (C), anti-HBs ≥10 mUI/ml (D) and anti-HEV IgG (E) by age groups in different regions of Peru.

**Fig 2:**
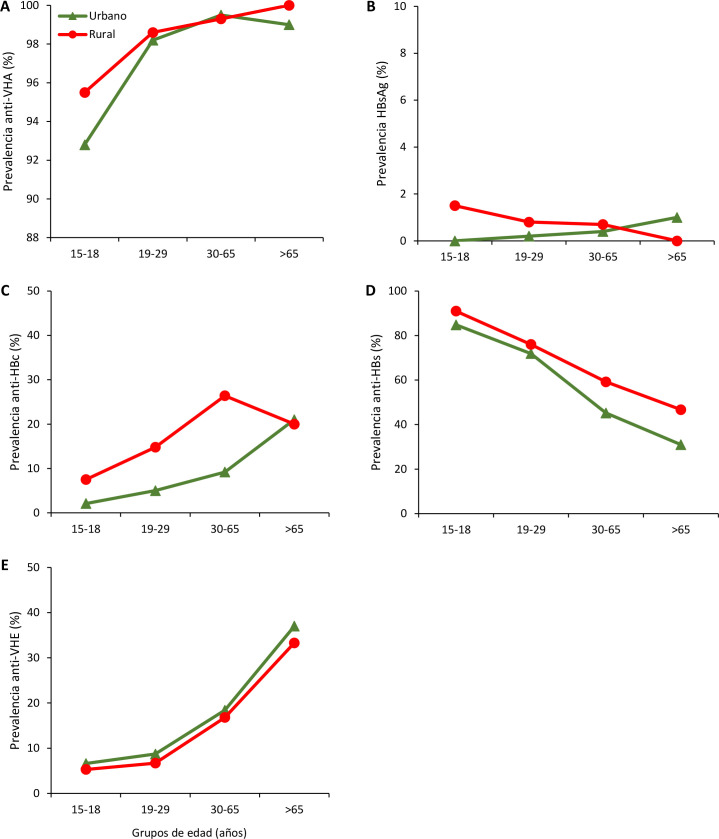
Prevalence rates of anti-HAV IgG (A), HBsAg (B), anti-HBc IgG (C), anti-HBs ≥10 mUI/ml (D) and anti-HEV IgG (E) by age groups in urban and rural areas of Peru.

**Fig 3:**
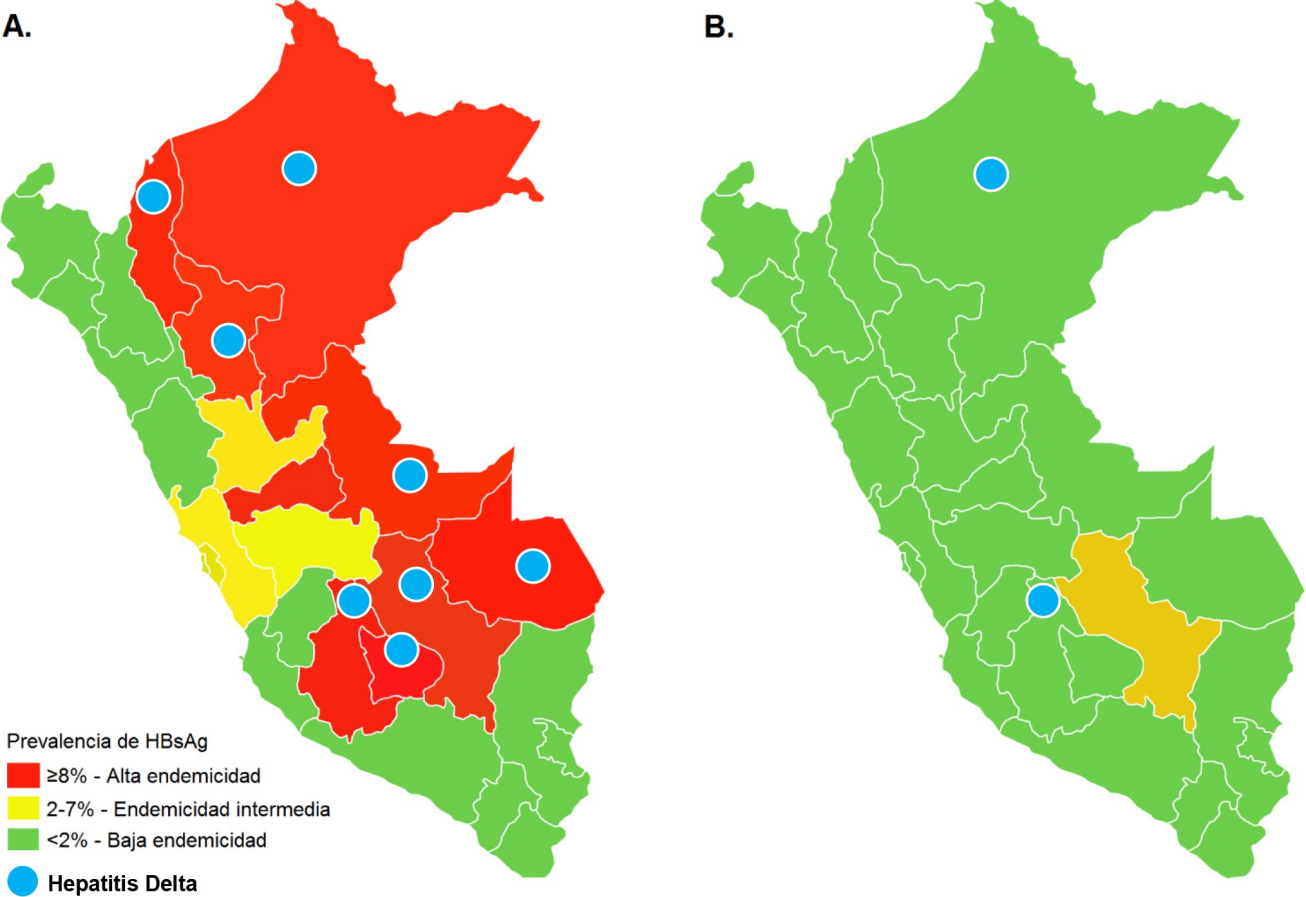
Prevalence of HBsAg and hepatitis Delta before (A) [19] and after the implementation of the hepatitis B vaccination program in Peru (B).

**Fig 4:**
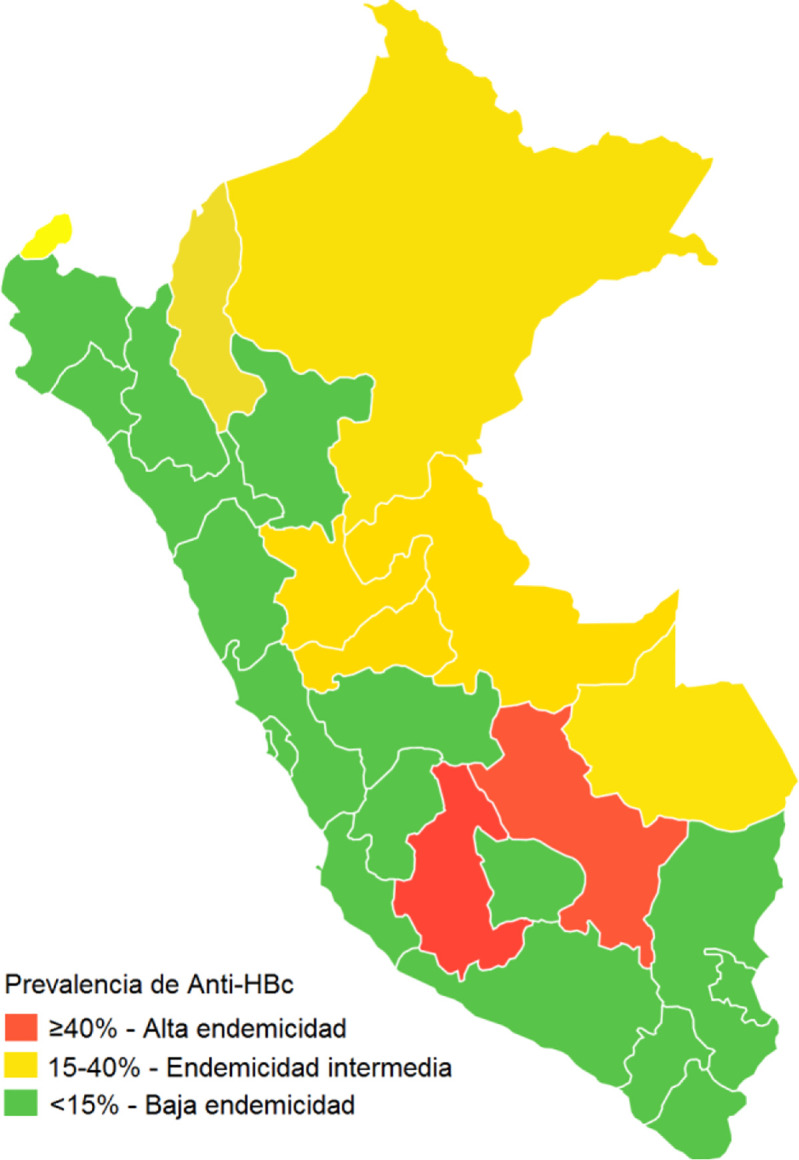
Prevalence of anti-HBc IgG after the implementation of the hepatitis B vaccination program in Peru.

**Fig 5:**
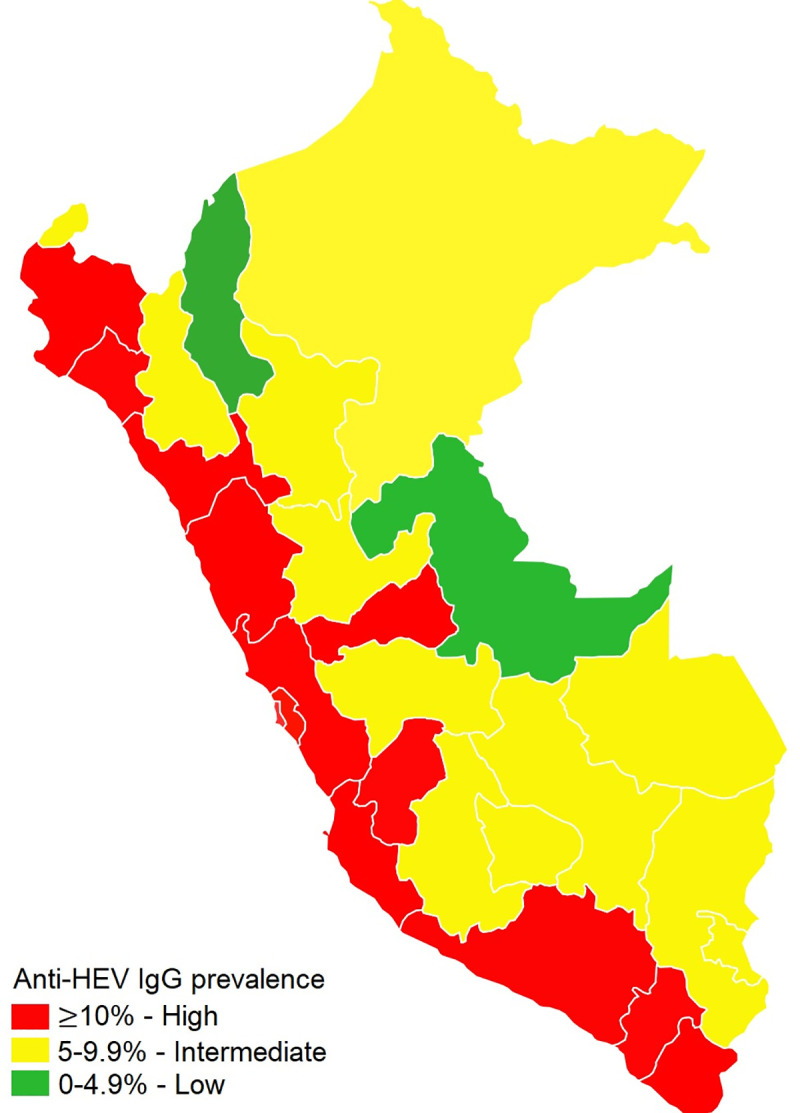
Prevalence of anti-HEV IgG in Peru, 2014–2015.

The publisher apologizes for the errors.
