# Correction: Correction: Seroepidemiology of hepatitis A, B, C, D and E virus infections in the general population of Peru: A cross-sectional study

**DOI:** 10.1371/journal.pone.0251539

**Published:** 2021-05-06

**Authors:** The PLOS ONE Staff

There are errors in the Correction published on April 8, 2021. The corrected figures are incorrect. The correct figures are:

**Fig 1:**
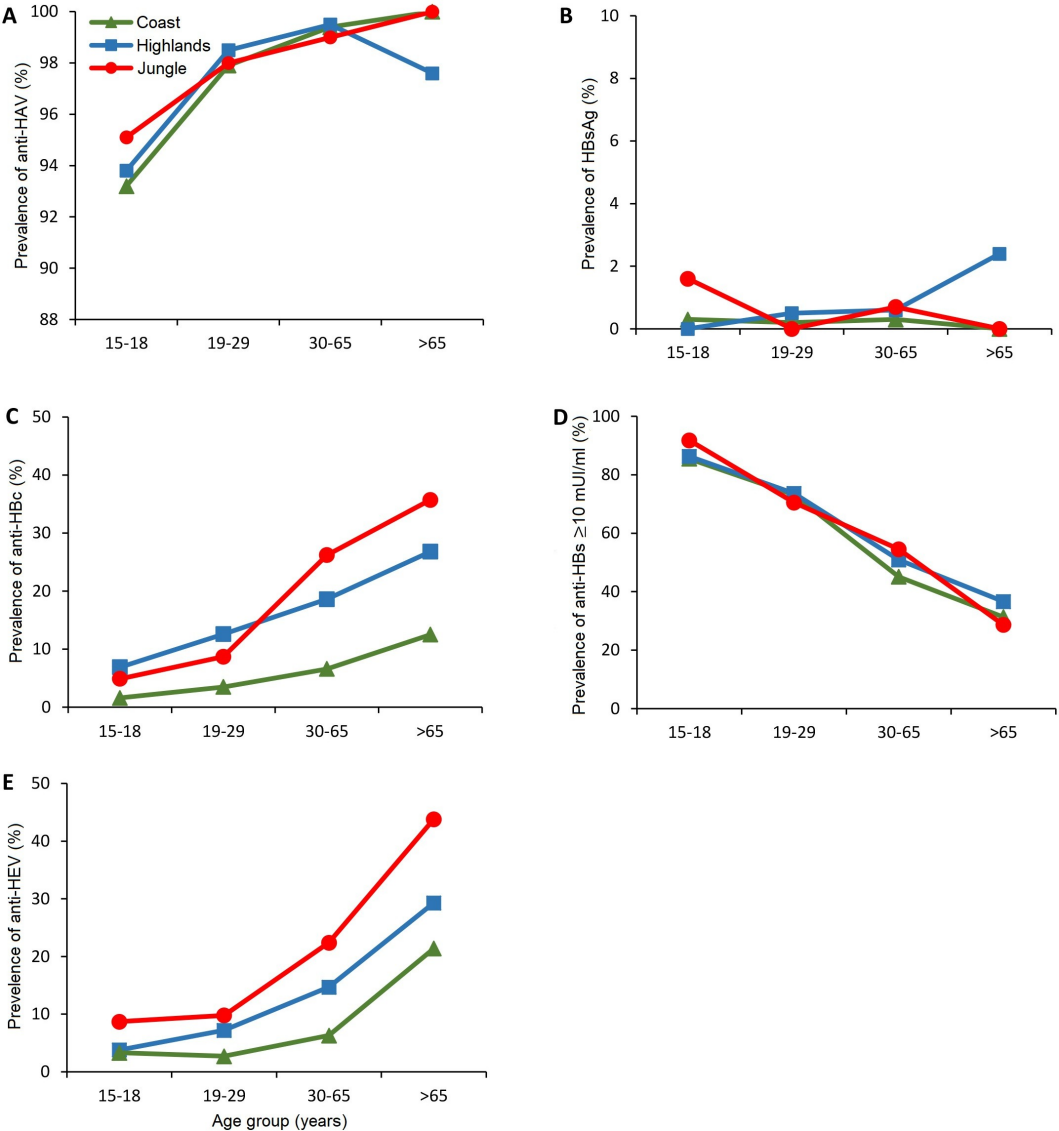
Prevalence rates of anti-HAV IgG (A), HBsAg (B), anti-HBc IgG (C), anti-HBs ≥10 mUI/ml (D) and anti-HEV IgG (E) by age groups in different regions of Peru.

**Fig 2:**
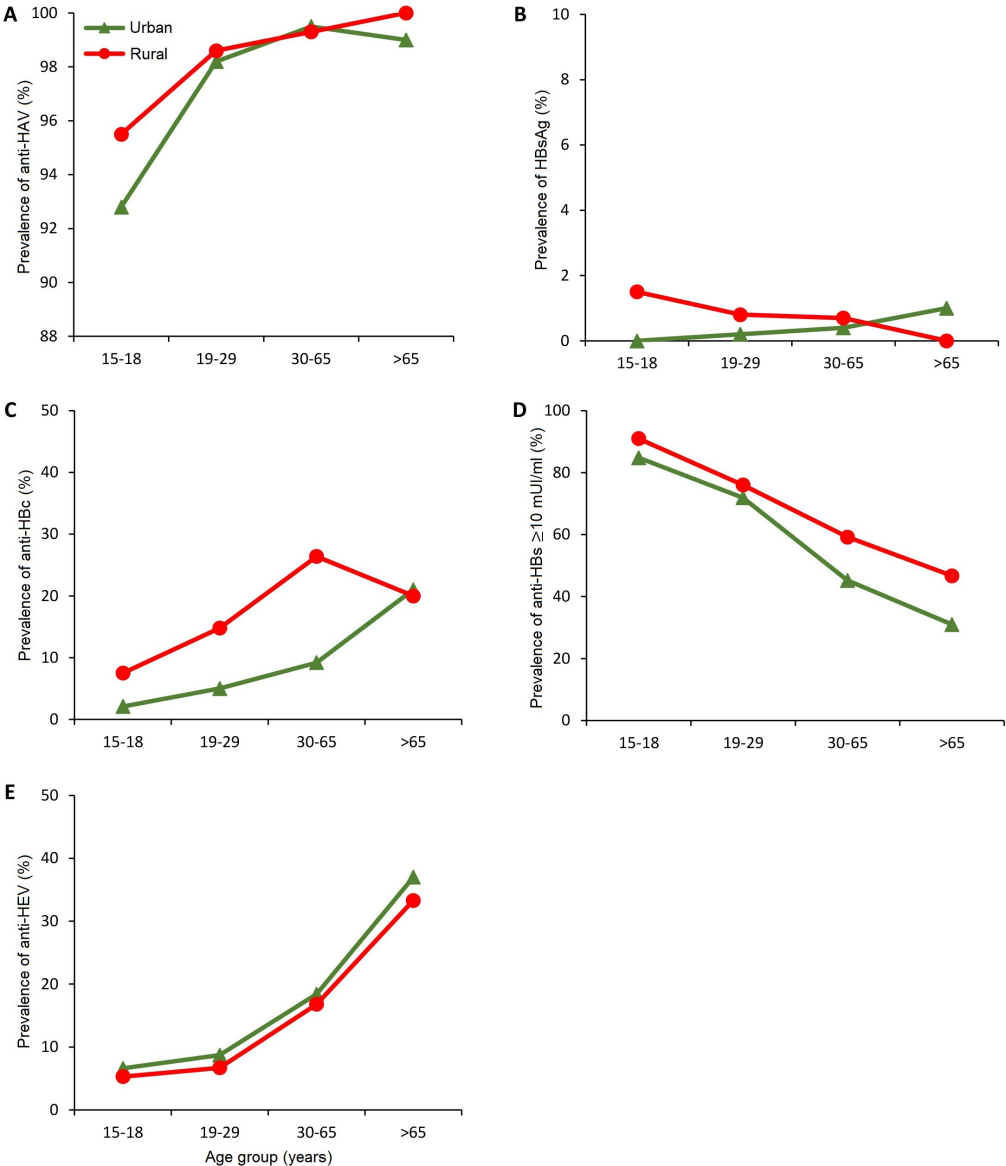
Prevalence rates of anti-HAV IgG (A), HBsAg (B), anti-HBc IgG (C), anti-HBs ≥10 mUI/ml (D) and anti-HEV IgG (E) by age groups in urban and rural areas of Peru.

**Fig 3:**
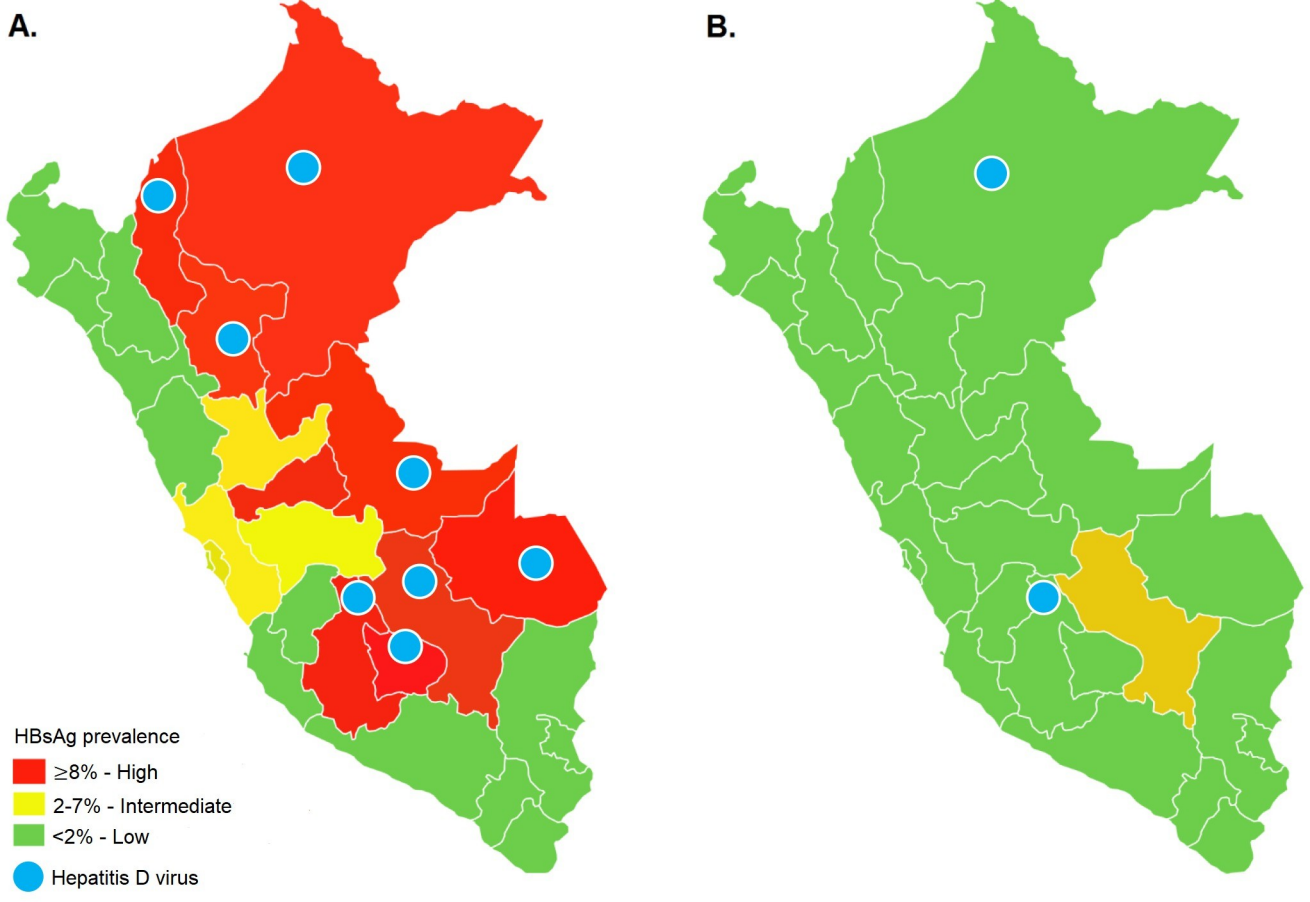
Prevalence of HBsAg and hepatitis Delta before (A) [19] and after the implementation of the hepatitis B vaccination program in Peru (B).

**Fig 4:**
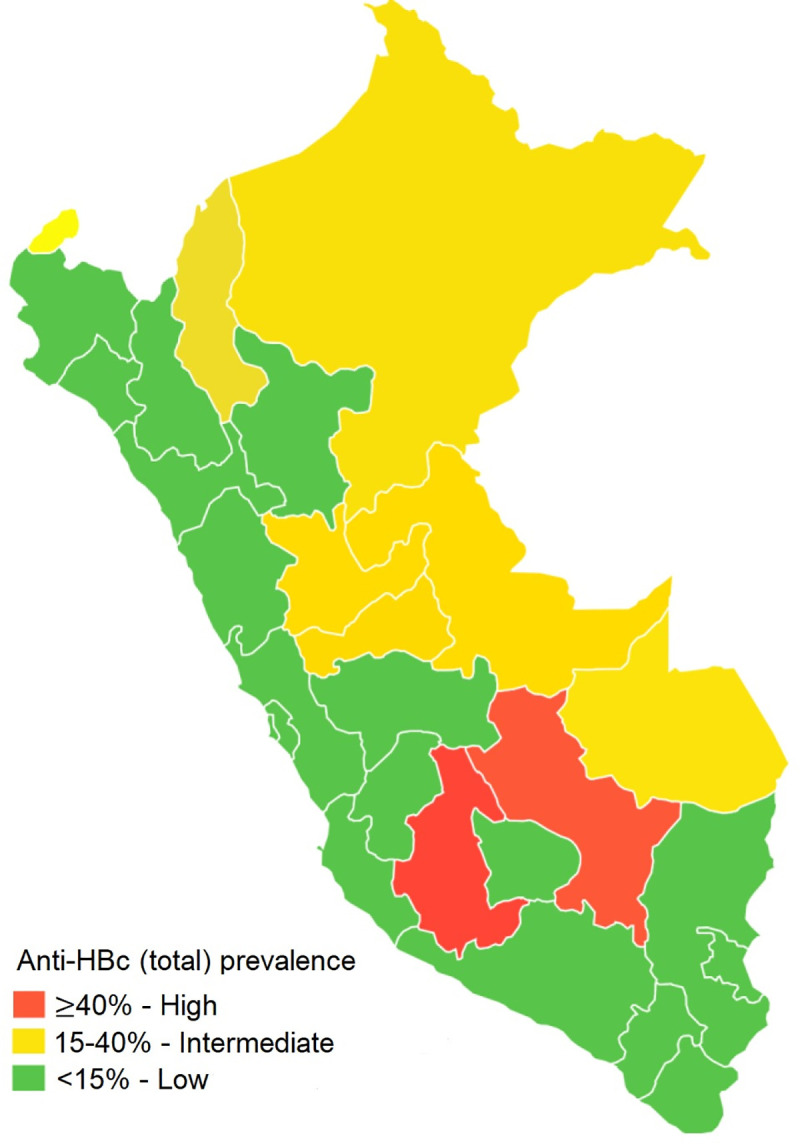
Prevalence of anti-HBc IgG after the implementation of the hepatitis B vaccination program in Peru.

**Fig 5:**
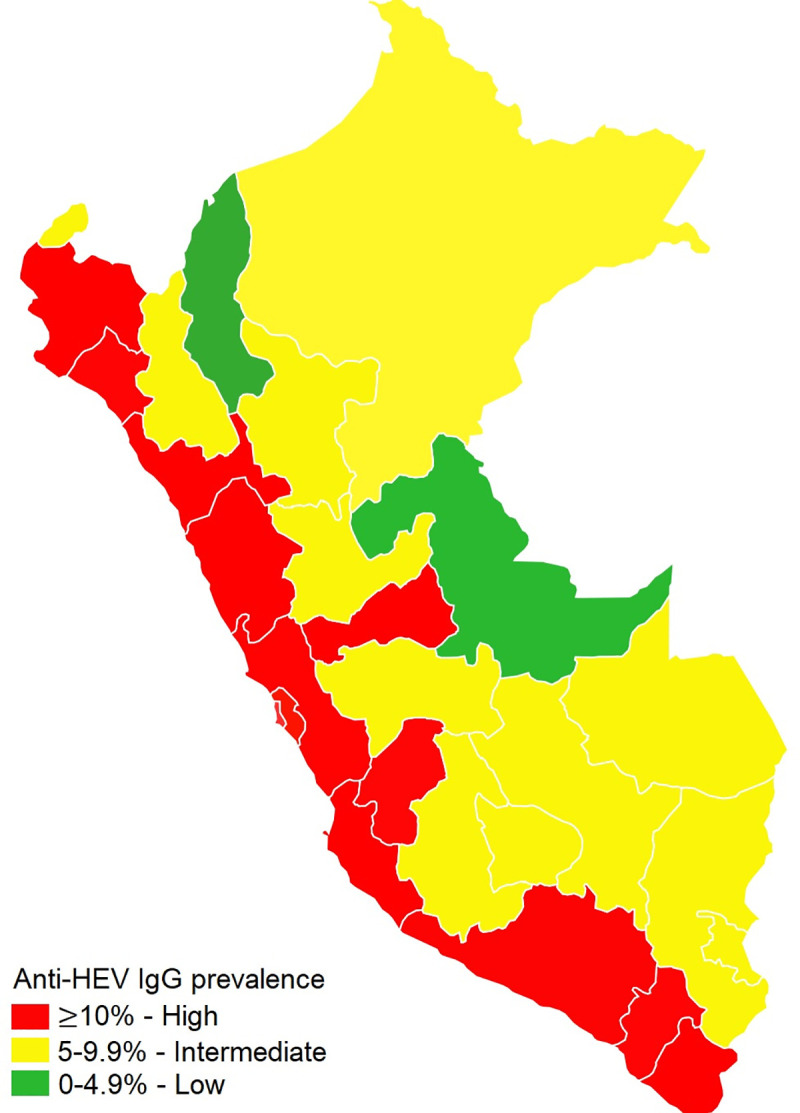
Prevalence of anti-HEV IgG in Peru, 2014–2015.

The publisher apologizes for the errors.
